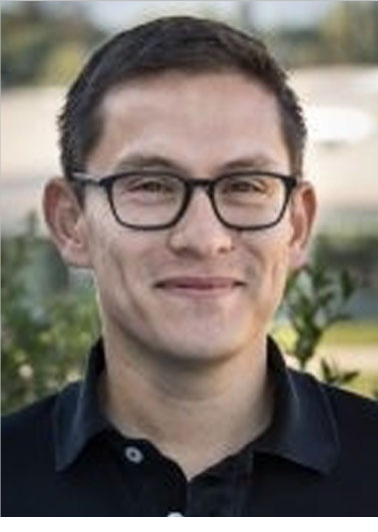# Genetically Encoded Activators of Small Molecules for Imaging and Drug Delivery

**DOI:** 10.1002/anie.201915521

**Published:** 2020-02-28

**Authors:** Zacharias Thiel, Jade Nguyen, Pablo Rivera‐Fuentes

**Affiliations:** ^1^ Institute of Chemical Sciences and Engineering EPF Lausanne CH C2 425, Station 6 1015 Lausanne Switzerland; ^2^ Laboratory of Organic Chemistry ETH Zurich Vladimir-Prelog-Weg 3 8093 Zurich Switzerland

**Keywords:** bioorthogonal labeling, biosensors, drug delivery, fluorescent probes, genetically encoded tags

## Abstract

Chemical biologists have developed many tools based on genetically encoded macromolecules and small, synthetic compounds. The two different approaches are extremely useful, but they have inherent limitations. In this Minireview, we highlight examples of strategies that combine both concepts to tackle challenging problems in chemical biology. We discuss applications in imaging, with a focus on super‐resolved techniques, and in probe and drug delivery. We propose future directions in this field, hoping to inspire chemical biologists to develop new combinations of synthetic and genetically encoded probes.

## Graphical abstract

**Big and small**: Genetically encoded activators and small synthetic probes can be used in combination to tackle complex problems in chemical biology. In this Minireview, a few applications in imaging, with a focus on super‐resolved techniques, and in probe and drug delivery, are highlighted. Future challenges in this field are also discussed.

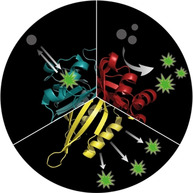

## 1 Introduction

One of the goals of chemical biology is to develop tools to study biological processes in living systems. Most of these tools can be categorized as either small molecules or genetically encoded, macromolecular probes. Both categories have their own advantages and limitations, and both are widely employed individually. In this Minireview, we highlight examples of chemical biology strategies that combine the two types of probes to alleviate the limitations of each other or to generate entirely new functions. We primarily discuss small molecules that are activated (converted to a fluorescent or biologically active state) by genetically encoded activators (GEAs). Other strategies that use small‐molecule ligands to modulate the function of proteins, in particular proteolysis targeting chimeras (PROTACs), have been recently reviewed elsewhere^1^, ^2^ and will not be covered here. Fluorogenic sensors based on chemical and enzymatic reactions have also been reviewed recently^3^, ^4^ and only a few examples will be discussed here.

**Figure 1:**
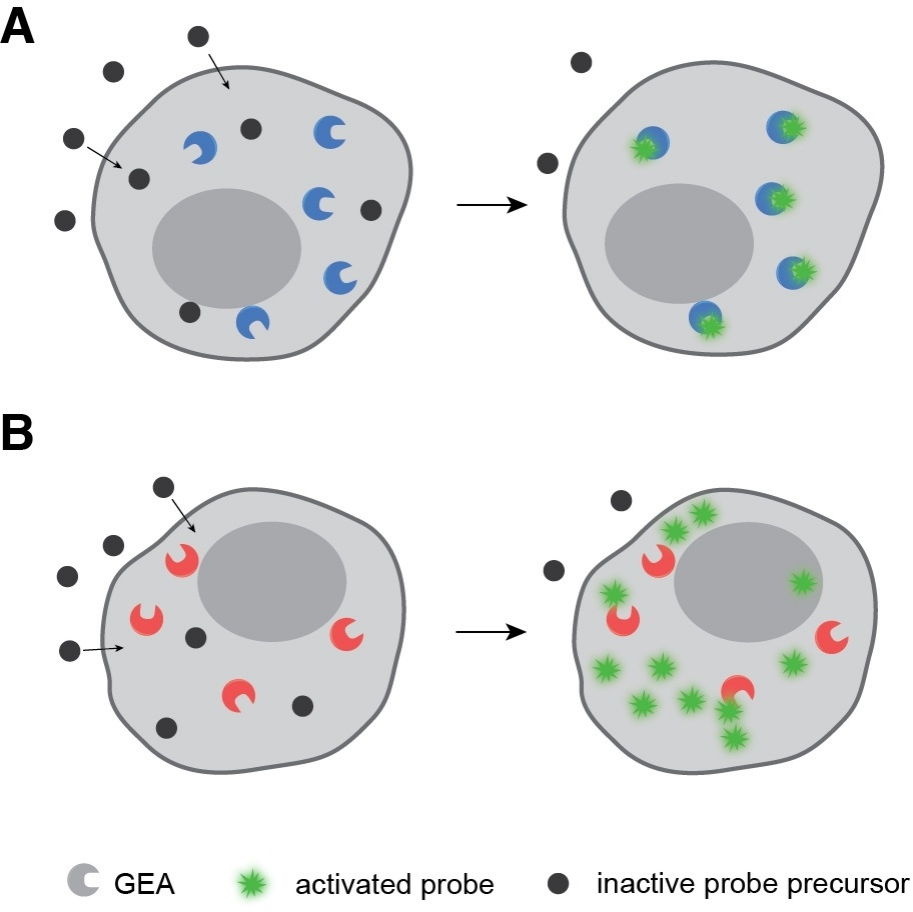
Schematic representation of the two different classes of GEAs discussed in this Minireview. A) Activation of precursor by binding. B) Enzymatic activation of precursors.

The first part of this Minireview discusses the activation of fluorogenic probes for live‐cell imaging, with a focus on super‐resolved techniques. These GEAs consist mostly of precursor‐binding peptides, proteins or nucleic acids (Figure 1 A) that activate externally supplied substrates by transient or covalent binding. The second part of this Minireview highlights examples of small‐molecule activation for controlling cellular process and drug delivery. These GEAs are enzymes that activate precursors without covalent binding (Figure 1 B). We provide an overview of various types of GEAs, compare them based on either their fluorogenicity (increase in fluorescence upon binding) or kinetics of probe activation, and mention some of their applications. We hope that by compiling some exciting developments in this field, this Minireview will inspire chemical biologists to develop new combinations of genetically encoded activators with small‐molecule reporters and actuators.

## 2 Genetically Encoded Activators for Imaging

Fluorescence imaging often relies on fluorescent proteins fused to the target of interest. These fusions are made by joining the genes of the target and the fluorescent protein within the same open reading frame. The resulting single chain of amino acids contains both proteins, often linked by a few amino acids. These genetic fusions provide excellent labeling specificity of the target of interest, which is a reason why this strategy is widely used. Small‐molecule fluorophores, however, offer complementary advantages. Their relatively small size perturbs their biological target less compared with proteins tags. Additionally, their photophysical properties, including brightness and photostability, are often superior to those of fluorescent proteins.^5^, ^6^ Furthermore, the properties of synthetic dyes can be fine‐tuned by rational chemical design, a feature that has proven to be much more challenging in fluorescent proteins. As a consequence, there is a broad selection of small‐molecule probes with vastly different properties and functions.

Ensuring that small‐molecule dyes target a specific macromolecule or subcellular location, however, remains a challenge. Some of the strategies available include the use of antibody–dye conjugates,^7^ chemical targeting units,^8^ short peptides,^9^, ^10^ and more recently, bioorthogonal ligation to proteins modified with non‐canonical amino acids.^11^, ^12^ Despite the success of these strategies, they are still hampered by either limited compatibility with living cells or lack of generalizability. An alternative approach to target small‐molecule probes is the use of GEAs. These proteins, peptides, or nucleic acids are present at the location of interest and interact selectively with probe precursors transforming them into an active form. Hence, they combine the excellent labeling specificity of protein tags with the superior properties and tunability of synthetic probes. A key requirement of this approach is the bioorthogonality of the GEAs and their substrates. In order to prevent non‐specific reactions of the probes, proteins or peptides that are not naturally expressed by the organism under study are usually employed. Thus, GEAs mainly comprise engineered proteins from bacterial or plant origin and have no mammalian homologues, which ensures bioorthogonality in mammalian systems.

Precursor‐binding GEAs can achieve highly specific labeling of intracellular structures for fluorescence imaging. These GEAs are designed to bind a probe precursor covalently or transiently, and the small molecules become active only when bound to the GEA. Hence, precursor‐binding GEAs are particularly useful for imaging and tracking experiments in which high contrast between bound and unbound species is required. In the case of fluorescence imaging, activation of the small molecule can be achieved by cleaving off a quenching unit, by modulating the photophysical properties of the dye upon binding, or by converting a pro‐fluorophore into a fluorescent compound.

**Figure 2:**
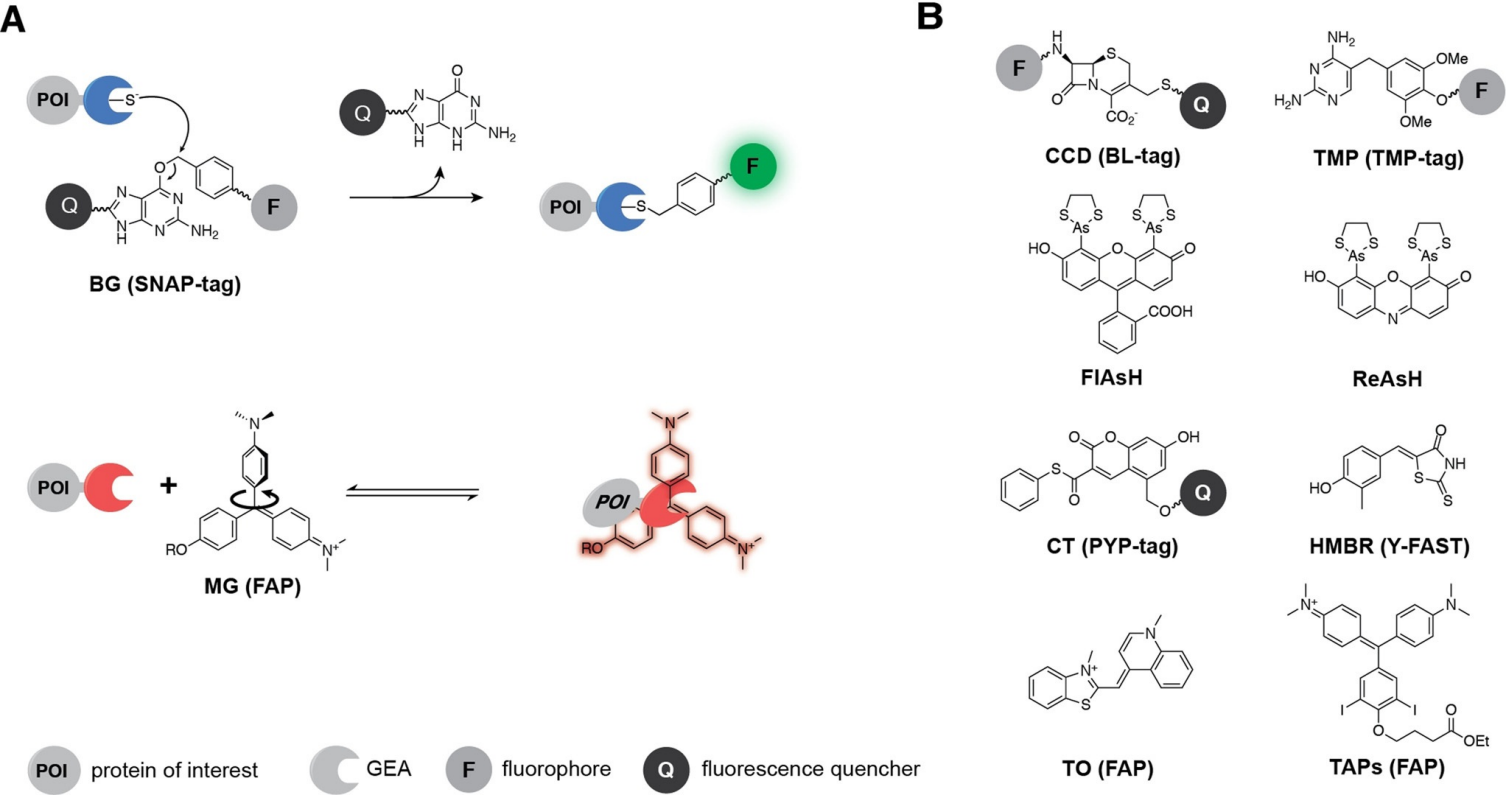
A) Schematic representation of precursor‐binding GEAs using selected examples. B) Structures of further substrates used for precursor‐binding GEAs, discussed in this Minireview.

The self‐labeling protein SNAP‐tag (20 kDa), developed by Johnsson and co‐workers in the early 2000s, covalently binds *O* ^6^‐benzylguanine (BG) conjugates and is one of the most prominent and commonly used protein tags.^13^ Even though these protein tags bind their substrates with high selectivity and are therefore bioorthogonal, their applicability is often compromised by residual unbound probe, which produces high background signal. Diminishing the background signal often requires tedious washing steps, which are oftentimes ineffective, and can be impractical to study fast biological processes. These drawbacks have been partially overcome by substrates that are modified with fluorescence quenching units, first reported by two different groups in 2011 (Figure 2 A).^14^, ^15^ These fluorophore–quencher pairs consist of a fluorophore that is connected via a short linker to a unit that quenches the fluorescence emission by Förster resonance energy transfer (FRET). During the labeling reaction, these quenching groups are cleaved off from the substrate and only the fluorophore remains bound to the protein, which results in up to 300‐fold increase in fluorescence intensity and allows for wash‐free labeling.

A similar example is the TMP‐tag, which was developed in the early 2000 s by Cornish and co‐workers.^16^ They engineered dihydrofolate reductase (18 kDa) from *Escherichia coli* to bind trimethoprim (TMP) conjugates with high affinity (Figure 2 B). Whereas probe binding to the first generation of TMP‐tag proteins was non‐covalent, further design of new substrates and engineering of the protein yielded tags that covalently bind TMP conjugates.^17^, ^18^ Special fluorophore–quencher pairs that release the fluorescence quenching unit upon binding to the GEA were developed for these tags.^19^ These substrates exhibit a 50‐fold increase in fluorescence intensity upon binding and can also be used for wash‐free fluorescence imaging of proteins.

Another example of fluorophore–quencher pairs is the bacterial beta‐lactamase (BL) tag (Figure 2 B), which was reported in the late 2000s.^20^ The hydrolytic activity of this small enzyme (29 kDa) is not found naturally in eukaryotic cells and its mechanism has been studied in detail,^21^ making it a good candidate for the development of a bioorthogonal GEA. Kikuchi and co‐workers reengineered the original BL by introducing a point mutation that results in covalent modification of the enzyme by a cephalosporin‐like substrate and simultaneous release of the quencher unit.^20^ Upon binding, the fluorescence intensity of these substrates increases approximately 30‐fold. The utility of this labeling approach was demonstrated using optimized substrates for pulse‐chase imaging experiments of the transmembrane trafficking of epidermal growth factor receptors.^22^

Binding of environment‐sensitive fluorophores is the second key concept of designing GEAs for fluorescence imaging applications. These fluorophores are characterized by very low fluorescence quantum yields in aqueous medium due to non‐radiative decay from the excited state via intramolecular rotation, aggregation‐induced quenching, or ground‐state isomerization.^23^ Binding to the protein tag greatly increases their fluorescence due to suppression of these non‐fluorescent states. Some of the most popular probes in this category were developed in the labs of Johnsson^24^, ^25^ and Lavis^26^, ^27^, ^28^ in the past few years. These probes are environment‐sensitive dyes that undergo fluorescence turn‐on upon binding to SNAP‐tag (20 kDa) or HaloTag (33 kDa)^29^ proteins. The emission wavelengths of these dyes span the whole region of visible light, their fluorescence intensities increase up to 1000‐fold upon binding,^30^ and they have been employed in numerous biological applications.

**Figure 3:**
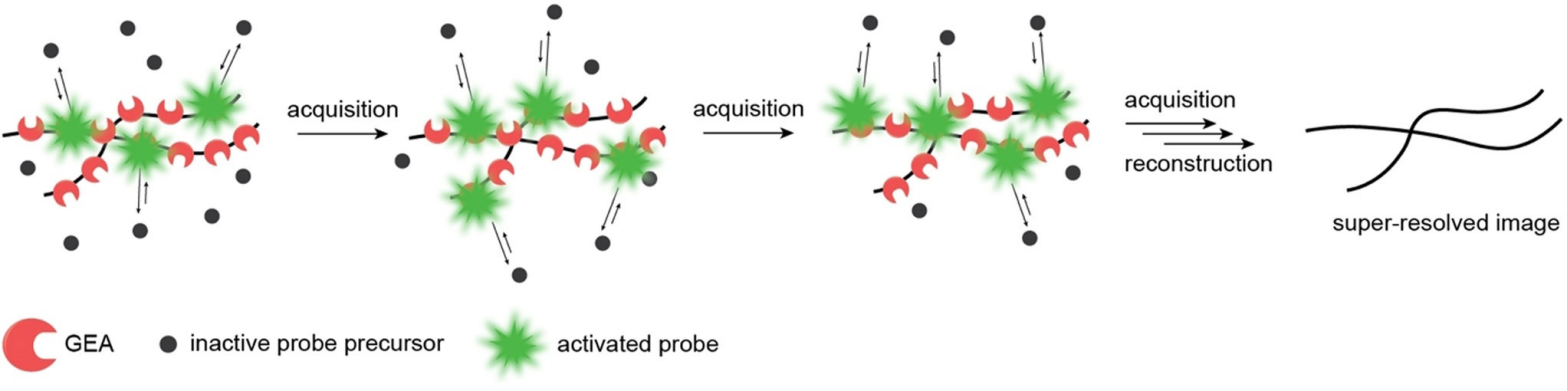
Schematic representation of the SMLM techniques based on transient binding of fluorogens.

Despite the success of environment‐sensitive SNAP‐tag and HaloTag dyes, other strategies are still worth exploring. Developed in the late 1990s, short peptide tags that bind the bisarsenical fluorogens FlAsH^31^ and ReAsH were some of the first GEAs reported (Figure 2 B).^32^ In this case, the fluorescence of the unbound dye is decreased by rotation around the aryl‐As bond. The As atoms can reversibly bind to a tetracysteine motif of a peptide tag, restricting intramolecular rotations for which a 2000‐fold increase in quantum yield is reported. Even though these systems suffer from background signal caused by non‐specific binding, their relatively small size (approximately 1 kDa, dye included) gives them an advantage over protein‐based GEAs like SNAP‐tag or HaloTag which are about 20 and 30 times larger, respectively. Furthermore, the transient binding of bisarsenical probes could be exploited for single‐molecule localization microscopy (SMLM, Figure 3). If the concentration of the fluorogen is chosen carefully, the fluorescence signals of individually activated molecules are sparse enough to allow for single‐molecule detection and reconstruction of super‐resolved images. This principle was first demonstrated using ReAsH, which could be imaged with nanometer precision on glass surfaces when binding to short peptide tags.^33^ Later, Zimmer and co‐workers used FlAsH for SMLM of cells that were infected with HIV bearing a short peptide tag, which did not affect the function of the sensitive viral capsid.^34^ As a result, the study of native viral replication in infected cells with unprecedented resolution was possible. Although SNAP‐tag and HaloTag have gained enormous popularity among biologists, smaller tags have the advantage of being less disruptive, in particular to small protein targets fused to them, and are worth exploring further.

Another GEA that activates environmentally sensitive fluorophores was derived from the photoactive yellow protein (PYP), a naturally occurring, small (14 kDa) photoreceptor protein found in purple bacteria.^35^, ^36^ In the late 2000s, Kikuchi and co‐workers designed a substrate based on a coumarin thioester (CT) linked to a fluorescein that binds to PYP (Figure 2 B). This construct is non‐fluorescent due to intramolecular association until the coumarin covalently binds to the PYP‐tag. Binding triggers the dissociation of the two fluorophores, which induces a 20‐fold fluorescence increase. Initially slow labeling kinetics were overcome by the development of improved substrates.^37^, ^38^ Reengineering of the protein tag further reduced the incubation time to just a few minutes.^39^ Also starting from PYP, Gautier and co‐workers developed the yellow fluorescence‐activating and absorption shifting tag (Y‐FAST) by directed evolution.^40^ These protein tags reversibly bind and activate 4‐hydroxy‐3‐methylbenzylidene‐rhodanine (HMBR, Figure 2 B), which has a structure that is closely related to that of the chromophore of green fluorescent protein (GFP). In unbound state, HMBR is almost non‐fluorescent due to intramolecular rotation, which is restricted when associated to the binding cavity of the protein. Binding increases the brightness of the probe by a factor of 1300. When bound, the chromophore is deprotonated, resulting in a considerable red‐shift in absorption and emission that further decreases background signal. The initially limited choice of available wavelengths was expanded by modifications of the structure of the chromophore.^41^ Due to the reversibility of binding, photobleached chromophores are constantly replaced by new molecules. Consequently, the apparent photobleaching is greatly reduced.^42^ This dye recycling mechanism opens up the possibility for acquiring super‐resolved images based on intensity fluctuations^43^ and perform single‐particle tracking analyses over long periods of time without loss of signal.^44^

Fluorogen‐activating proteins (FAPs) are single‐chain variable fragment antibodies (approximately 14 kDa) that were developed in the late 2000s to bind analogues of malachite green (MG, Figure 2 A) and thiazole orange (TO, Figure 2 B).^45^, ^46^ Emission wavelengths above 600 nm and up to 18 000‐fold increase in fluorescence intensity upon binding make these systems attractive for live‐cell fluorescence microscopy experiments. Despite these advantageous photophysical properties, imaging using FAP genetically fused to specific proteins was initially restricted to proteins on the cell surface and the secretory system. First‐generation FAPs contained many internal disulfide bridges, which led to misfolding and loss of fluorogen‐binding capability in reducing environments. This limitation was overcome using directed evolution. FAPs that also remain functional under reducing conditions were developed and successfully targeted to the cytosol, endoplasmic reticulum, mitochondria, and the nucleus.^47^, ^48^, ^49^ Further developments of the fluorogen–FAP pairs allowed for specific control over cell permeability,^50^, ^51^ excitation and emission wavelengths,^52^ and selective targeting in live animals.^50^ The high contrast and transient nature of binding makes FAPs amenable for SMLM. Bruchez and co‐workers reported the application of FAPs for binding and activation localization microscopy (BALM), which is closely related to the now more popular technique known as point accumulation for imaging in nanoscale topography (PAINT).^53^ The high photostability of FAP–chromophore pairs makes them well suited for particle tracking. Moerner and co‐workers demonstrated this advantage by tracking single proteins of lysozyme homologue SpmX fused to FAP in living Caulobacter cells.^51^ Lidke, Bruchez, and co‐workers used FAPs for single‐protein tracking to visualize FcϵRI receptor dynamics.^54^ The high selectivity of FAPs towards their probes and their tunability broadened the scope of applications beyond fluorescence imaging. Derivatives of MG modified with heavy atoms were used in combination with FAPs for the light‐triggered production of reactive oxygen species (ROS).^55^ When bound to FAPs and irradiated, these targeted and activated photosensitizers (TAPs, Figure 2 B) induced the formation of singlet oxygen due to intersystem crossing. Excitation wavelengths in the near infrared makes these probes especially useful for cell ablation in multicellular organisms in which high optical tissue penetration is required. This application was demonstrated in adult zebrafish where light mediated production of singlet oxygen was used for cardiac cell ablation.^55^

Beyond the realm of proteins, nucleic acids can also be used to activate otherwise non‐fluorescent probes. In the early 2010s, Jaffrey and co‐workers developed Spinach, an RNA aptamer that binds a GFP‐chromophore analogue, thereby increasing the brightness of the fluorophore by a factor of 2000 and enabling live‐cell imaging of RNA.^56^ Further improvements in both the aptamer and the small molecule have delivered a wide selection of tools for RNA imaging.^57^, ^58^, ^59^, ^60^ In a related strategy, Palmer and co‐workers used an aptamer that binds and deactivates a cobalamin quencher to create a platform for multicolor RNA imaging with excellent signals.^61^ These tools have enabled imaging RNA in living cells, in some cases employing super‐resolution techniques.^60^

**Figure 4:**
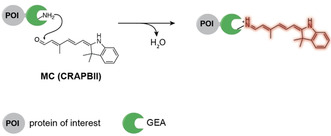
Schematic representation of the conversion of a merocyanine dye precursor into a fluorophore by CRABPII.

GEAs that convert a functional group of the small molecule upon binding are less common, but a few examples exist. Inspired by the naturally occurring conversion of retinal into a protein‐bound chromophore, Borhan and co‐workers engineered a cellular retinoic acid binding protein II (CRABPII, 16 kDa, Figure 4) in 2015.^62^ This protein binds and activates a merocyanine dye precursor through a nucleophilic attack of a lysine residue in its active site. An iminium species with push–pull character and an extended conjugated π‐system is formed. The quantum yield of this protein‐bound iminium species is 10 times larger than that of the precursor. Since this system was not compatible with expression in mammalian cells and thus limited to imaging bacteria, the same group recently engineered a human cellular retinol binding protein that activates the same probe precursor and can be expressed in mammalian cells.^63^ Similarly, Arnold and co‐workers used directed evolution to develop a microbial rhodopsin (28 kDa) to bind a merocyanine dye with a prolonged polyene chain, which enabled emission wavelengths above 770 nm.^64^ Even though the fluorescence increase of these systems is not as large as other examples highlighted in this Minireview, they are substantially brighter than fluorescent proteins that emit at comparable wavelengths.

## 3 Genetically Encoded Activators for Probe and Drug Delivery

The GEAs mentioned in the previous section are very useful for imaging applications because they form a covalent bond or a strong supramolecular interaction with the pro‐fluorophore. This strong interaction allows for localization of the GEA, and the target fused to it, using the signal emitted by the activated fluorescent probe. When the goal is not imaging, but rather triggering a specific biological response, probes need to be able to diffuse away from the activator to exert their effects. A notable exception is the interrogation of the function of the same protein that is labeled with a GEA, for example using the REX^65^ or BOLT^66^ technologies. In most cases, however, activated probes need to be available as freely diffusing molecules in high local concentrations. This kind of activation can be achieved by using enzymes that are able to transform inactive substrates into active probes with high specificity and large turnover numbers. Typically, natural or engineered enzymes that are not naturally expressed by the organism of interest are used as GEAs to create bioorthogonal enzyme‐substrate pairs. This method is similar to the widely employed reporter gene technologies developed in the 1990s,^67^ which use β‐galactosidase, luciferase, or GFP to visualize gene expression. The methods using GEAs to improve the selectivity of antitumor agents are known as gene‐directed‐enzyme prodrug therapy (GDEPT) and virus‐directed‐enzyme prodrug therapy (VDEPT), and were also developed in the 1990s.^68^

**Figure 5:**
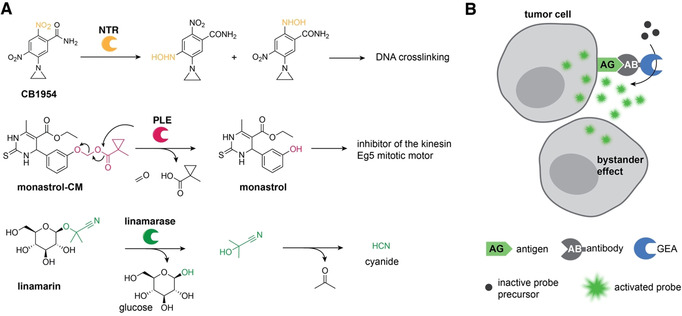
A) Schematic representation of three types of enzymatically activatable GEAs using selected examples. B) Schematic representation of the antibody‐directed enzyme prodrug therapy (ADEPT).

Nitroreductases (NTR) are enzymes capable of reducing nitroaromatic groups into their corresponding anilines.^69^ Bacterial nitroreductases are well characterized and their activity is exploited for the activation of nitro‐substituted substrates^70^, ^71^, ^72^, ^73^ by wild‐type NTR from *Escherichia coli*.^74^ Nitroreductase activity is also present in mammalian cells, but the enzymes responsible for the reduction of nitro groups have not been identified yet.^75^ Nevertheless, the increased nitroreductase activity of solid tumors is used for tumor‐specific fluorophore activation^76^ and activation of pro‐drugs in cancer therapy.^77^ Strict bioorthogonality is not ensured because NTR activity from endogenous enzymes is also observed in healthy mammalian cells, but employing bacterial or engineered NTRs can drastically increase the activation efficiency of nitroaromatic probes above the background of endogenous enzymes. Examples of this strategy include the activation of the DNA cross‐linking pro‐drug CB1954 (Figure 5 A)^78^ by wild‐type NTR with catalytic efficiency (*k*_cat_/K_m_) in the range of 7×10^3^ m ^−1^ s^−1^ and the use of an engineered variant of the bacterial NTR for cell‐specific delivery of various small molecules in mammalian cells.^79^ Mutant NTRs can activate substrates such as CB1954 and nitrofurazone with up to 6‐fold increase in catalytic efficiency (*k*_cat_/K_m_=4.3×10^4^ m ^−1^ s^−1^) compared to wild‐type NTR.^74^

In the 2010s, the Lavis group developed a selective ester–esterase pair for mammalian cells based on sterically hindered esters and an orthogonal esterase from porcine liver (PLE) that can hydrolyze a fluorescein di(1‐methylcyclopropanecarboxy‐methyl ether) substrate with *k*_cat_/K_m_=5.1×10^4^ m ^−1^ s^−1^.^80^ Several substrates, including a precursor of the kinesin inhibitor monastrol were activated in various mammalian cell types transfected with the exogenous gene of PLE with little activation of the probes by endogenous esterases. This work demonstrates that the cleavage of bioorthogonal esters can be used for the delivery of bioactive or fluorescent compounds with hydroxy groups (Figure 5 A). Recently, Dickinson and co‐workers used a split BS2 esterase that only becomes active upon protein–protein interaction.^81^ BS2 can hydrolyze the model substrate *p*‐nitrophenyl acetate with a catalytic efficiency of about *k*_cat_/K_m_=10 m ^−1^ s^−1^.^82^ This system was used to release ester‐protected fluorophores as well as bioactive compounds. A remarkable novel feature of this GEA is its dependence on a protein–protein interaction, which can be used for on‐demand activation of substrates. A different bioorthogonal deprotection strategy of hydroxy groups was developed by Meggers, Reetz and co‐workers,^83^ who reported an engineered bacterial cytochrome p450 fatty acid hydrolase that selectively cleaves off a propargylic ether coumarin‐derived substrate with *k*_cat_/K_m_=76 m ^−1^ s^−1^.

Linamarase from cassava was the first plant enzyme used as GEA in mammals, as part of a gene‐therapy project, in the late 1990s.^84^ Linamarase hydrolyzes the non‐toxic linamarin into glucose, acetone, and highly toxic cyanide (Figure 5 A). The catalytic efficiency of the hydrolysis of linamarin by linamarase calculated from reported data is 7.6 m ^−1^ s^−1^.^85^ The enzyme was expressed in rat cells and was able to eradicate brain tumors in live animals. This system is particularly efficient for tumor ablation because cyanide is able to cross cell membranes. Consequently, significant toxicity is exerted on neighboring cells that do not express linamarase as a consequence of the bystander effect.^84^ However, this technique requires artificial, invasive gene transfer into the cells or organisms of interest.

GEAs can also be combined with antibodies to target them to a specific cell type. This concept, which was already introduced in the 1990s, is known as antibody‐directed enzyme prodrug therapy (ADEPT). For example, tumor‐associated monoclonal antibodies are linked to the GEA to target antigens of tumor tissues (Figure 5 B).^68^ An inactive precursor is then converted by the pre‐targeted enzyme localized on the tumor surface into a toxic agent. This method is especially useful due to the many possible combinations of bioorthogonal enzymes and targeting antibodies.^86^

## 4 Conclusion and Outlook

GEAs comprise a family of macromolecules that can be used to activate small‐molecule probes in live cells employing various modes of action. They offer excellent targeting specificity up to the level of single molecules. Moreover, the small synthetic probes can be easily tuned to have a vast variety of effects. Hence, GEAs combine the benefits of both genetically encoded targets and synthetic small molecules.

Even though there is already a considerable selection of GEA–small‐molecule pairs available, there is still a great potential for further development. A remaining challenge lies in improving the bioorthogonality of the reactions. Starting points for engineering GEAs are bacterial or plant proteins that do not have an analogous counterpart in the organism of interest. However, greater bioorthogonality could be achieved by introducing de novo designed proteins and enzymes with orthogonal reactivities. Examples of this strategy are an artificial retro‐aldolase that can activate an unnatural substrate to give a fluorescent reporter in eukaryotic as well as prokaryotic cells,^87^, ^88^ a recently reported designer β‐barrel‐structured protein capable of transiently binding and activating a GFP‐like fluorogen,^89^ and the use of artificial metalloenzymes to activate synthetic probes.^90^ Despite these recent developments, there is still plenty of room for improvement in terms of photophysical properties, binding affinities, or turnover numbers to make these strategies broadly applicable.

Furthermore, the implementation of fluorogenic substrates that feature functionalities beyond simple turn‐on fluorescence could lead to the development of sophisticated systems for fluorescence microscopy. A recent example is the development of a genetically targetable pH sensor that is based on FAPs.^91^ Currently, all GEAs that were developed for SMLM techniques are based on transient binding and activation events. To prevent overlap of signals and enable localization of single molecules, labeling density and binding affinity have to be meticulously fine‐tuned. GEAs based on spontaneously blinking^92^ or fluxional fluorophores^93^ would allow for high labeling density regardless of binding affinity and thus could impact studies that rely on single‐molecule localization techniques.

There is already a large selection of methods for activating small‐molecule probes using GEAs. Challenges in this area, in particular for therapeutic applications, are related to the delivery of the GEA itself to the target of interest. Advances in antibody‐enzyme conjugates and transduction methods based on biocompatible polymers^94^ or nanocapsules^95^ are progressing in this direction.

Finally, GEAs that can convert endogenous small molecules into other bioactive compounds are particularly interesting. Recently, the Mootha lab developed GEAs to modulate the intracellular ratios of NADH to NAD^+[96]^ and NADPH to NADP^+^.^97^ These tools enabled the study of redox imbalances within subcellular locations based on the modulation of these redox buffers. In this direction, novel GEAs could be designed to either generate or consume other biologically important small molecules that cannot be directly encoded in the genome. These tools would facilitate the study of the subcellular compartmentalization of metabolism and signaling, as well as the creation of new functions in synthetic biology. The generation of these tools will require new creative solutions that combine small‐molecule and genetically encoded probe development.

## Conflict of interest

The authors declare no conflict of interest.

## Biographical Information

*Zacharias Thiel obtained his BSc degree in technical chemistry at the Vienna University of Technology. After a five‐month internship at the Fraunhofer Institute for Environmental, Safety, and Energy Technology, he completed his MSc degree in chemistry at ETH Zurich. Since 2017, he has been PhD student at ETH Zurich and EPF Lausanne, working on the development and implementation of single‐molecule fluorescent sensors of enzymatic activity*.

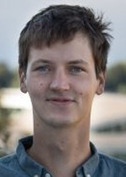

## Biographical Information

*Jade Nguyen obtained her BSc degree in chemistry and chemical engineering at EPF Lausanne in 2015. During her Bachelor studies, she spent one year at the Humboldt University in Berlin as an exchange student. She then completed her MSc degree in chemistry at ETH Zurich. Since 2017, she has been a PhD student at ETH Zurich and EPF Lausanne, working on enzymatically activatable probes leading to redox stress in live cells and elucidation of the induced biochemical pathways*.

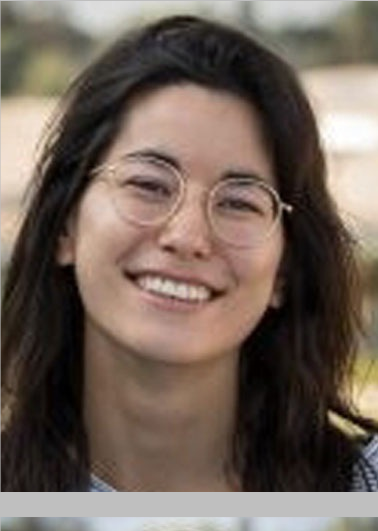

## Biographical Information

*Pablo Rivera‐Fuentes obtained his PhD from ETH Zurich in 2012. After postdoctoral appointments at MIT and the University of Oxford, he started his independent career at ETH Zurich in late 2015. Since 2019, he has been Assistant Professor of Chemical Biology at EPF Lausanne, working on the development of chemical tools to visualize and control biological processes in live cells*.